# Aquaporin‐4: Unraveling the Link Between Reactive Astrogliosis and Neuroinflammation in Down syndrome with Alzheimer’s Disease Pathogenesis

**DOI:** 10.1002/alz.092708

**Published:** 2025-01-03

**Authors:** Cherie A Stringer, Haley Miyasato, Kevin Camey, Phong Ngo, Elizabeth J. Andrews, Florence Lai, Milos D Ikonomovic, Julia Kofler, Sierra Wright, Michael J. Phelan, Elizabeth Head

**Affiliations:** ^1^ University of California, Irvine, Irvine, CA USA; ^2^ Massachusetts General Hospital, Harvard Medical School, Boston, MA USA; ^3^ University of Pittsburgh School of Medicine, Pittsburgh, PA USA; ^4^ VA Pittsburgh Healthcare System, Pittsburgh, PA USA; ^5^ Alzheimer Biomarker Consortium‐Down Syndrome, Irvine, CA USA

## Abstract

**Background:**

Down syndrome (DS) is characterized by the overexpression of the amyloid precursor protein gene (APP) and pro‐inflammatory genes, leading to progressive beta‐amyloid (Aβ) accumulation. This accumulation in DS may exacerbate neuroinflammation, contributing to the pathogenesis of Alzheimer’s Disease (AD). Experimental models suggest that aquaporin 4 (AQP4), an astrocytic water channel implicated in Aβ clearance, is mislocalized with increased Aβ burden. It is unclear whether the mislocalization of AQP4 is observed and connected with reactive astrogliosis in response to Aβ and the progression of AD in DS. We hypothesize AQP4 expression is increased and mislocalized from astrocytic endfeet, contributing to an astrocytic neuroinflammatory response in DS with AD pathogenesis.

**Methods:**

This study analyzed AQP4 distribution and expression via immunohistochemistry for AQP4 in 70 frontal (FR) and 64 occipital (OC) cortical human post‐mortem brain samples. Diagnostic classes included aged controls, DS, DS with AD, and sporadic AD (sAD). Semi‐quantitative analysis compared AQP4 distribution across diagnostic classes and brain regions. AQP4 expression was quantified using QuPath. Immunofluorescence (IF) assessed co‐localization and distribution of neuroinflammatory markers complement C3 (C3) and S100β with GFAP‐labeled astrocytes.

**Results:**

AQP4 immunolabeling was observed for expression, distribution, and astrocytic morphology. In DS with AD, AQP4 was expressed away from endfeet, indicating a higher prevalence of reactive astrocytic morphologies compared with DS and control groups. Two‐Way ANOVA analysis revealed increased AQP4 expression in DS with AD compared with DS in both FR (p<0.0001) and OC (p = 0.0007) regions. IF for C3 exhibited a plaque‐like distribution and S100β co‐localized with GFAP‐labeled astrocytes in DS with AD and sAD not seen in DS and controls.

**Conclusion:**

The observed increased expression and mislocalization of AQP4 from endfeet, coupled with heightened AQP4‐labeled reactive astrocytic morphology, supports our hypothesis that AQP4 is involved in AD pathology in DS. Altered neuroimmune marker distribution and co‐localization in DS with AD, compared to DS alone, further supports the hypothesis that AQP4 localization contributes to reactive astrogliosis. Subsequent investigations will explore correlations between AQP4 localization changes, Aβ levels, and increased expression of neuroinflammatory markers, including C3 and S100β.

**Funding**: NINDS (T32 NS121727‐01), ADRC (P30AG066519), ABC‐DS (U19AG068054), and Brightfocus (BFF17_0008).

